# Supplementary figures and images for: Coronavirus nucleocapsid protein enhances the binding of p-PKCα to RACK1: Implications for inhibition of nucleocytoplasmic trafficking and suppression of the innate immune response
Source: PLoS Pathog. 2024 Nov 27;20(11):e1012097. doi: 10.1371/journal.ppat.1012097 (PMC11633972; doi:10.1371/journal.ppat.1012097)

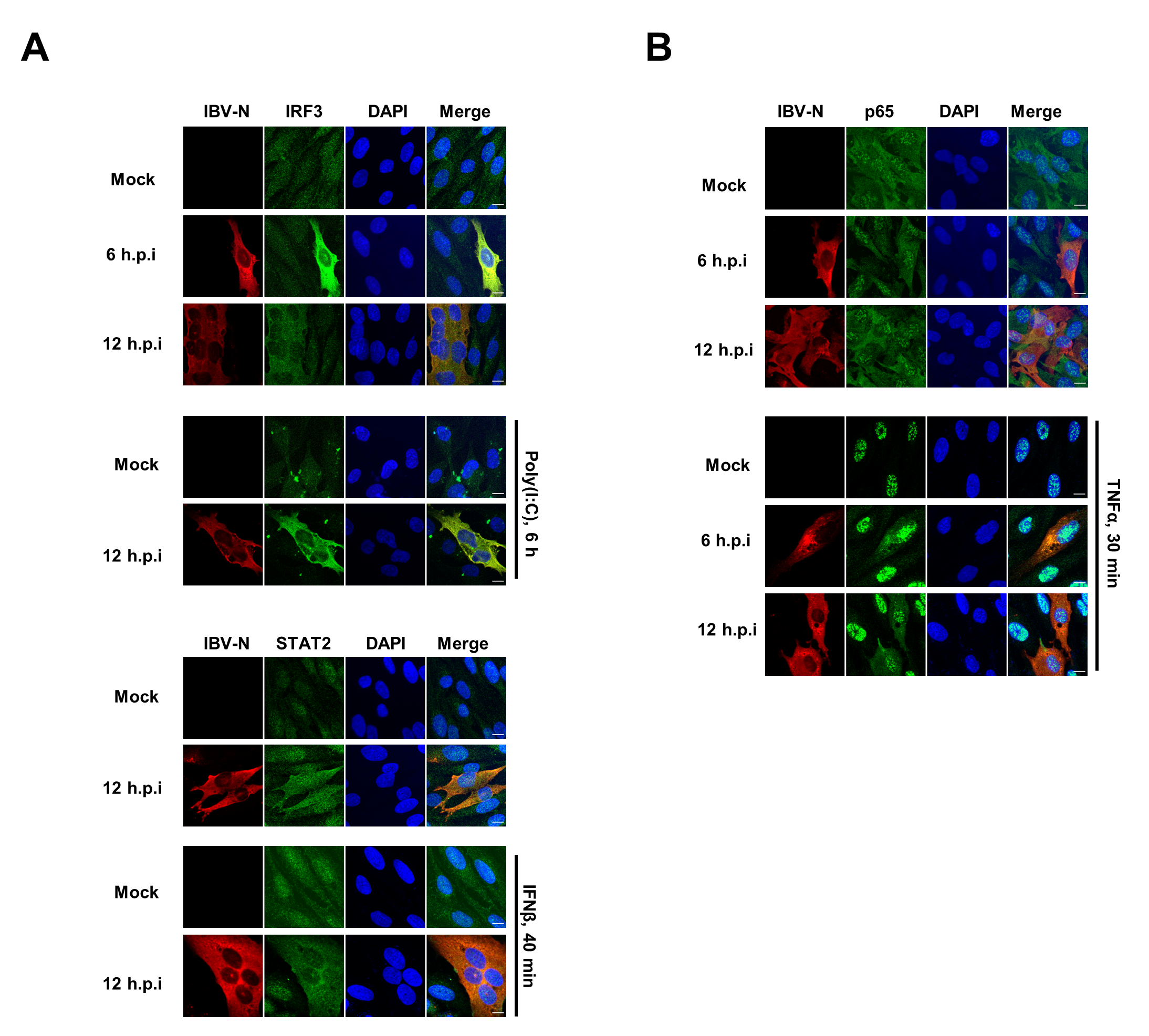

Supplement: S1 Fig — (A-B) DF-1 cells were infected with IBV at an MOI of 1, followed by treatment with poly(I:C), IFNβ, or TNFα. Cells were harvested at the indicated time points and subjected to immunofluorescence analysis. Representative images from three independent experiments are shown. Scale bars: 10 μm. (TIF) [file ppat.1012097.s001.tif]

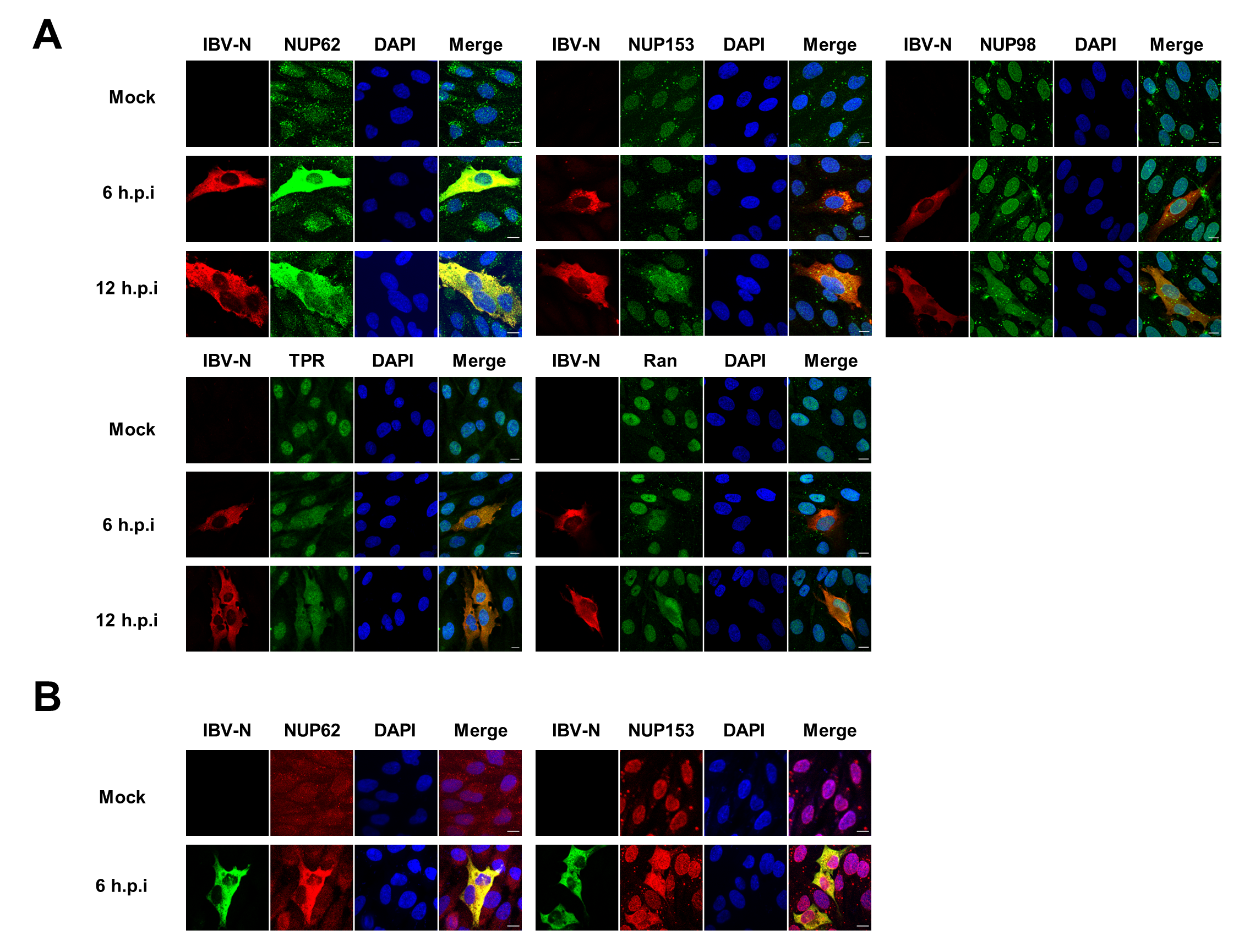

Supplement: S2 Fig — (A-B) DF-1 cells were infected with IBV at an MOI of 1 or mock-infected, harvested at the indicated time points, and subjected to immunofluorescence analysis. Representative images from three independent experiments are shown. Scale bars: 10 μm. (TIF) [file ppat.1012097.s002.tif]

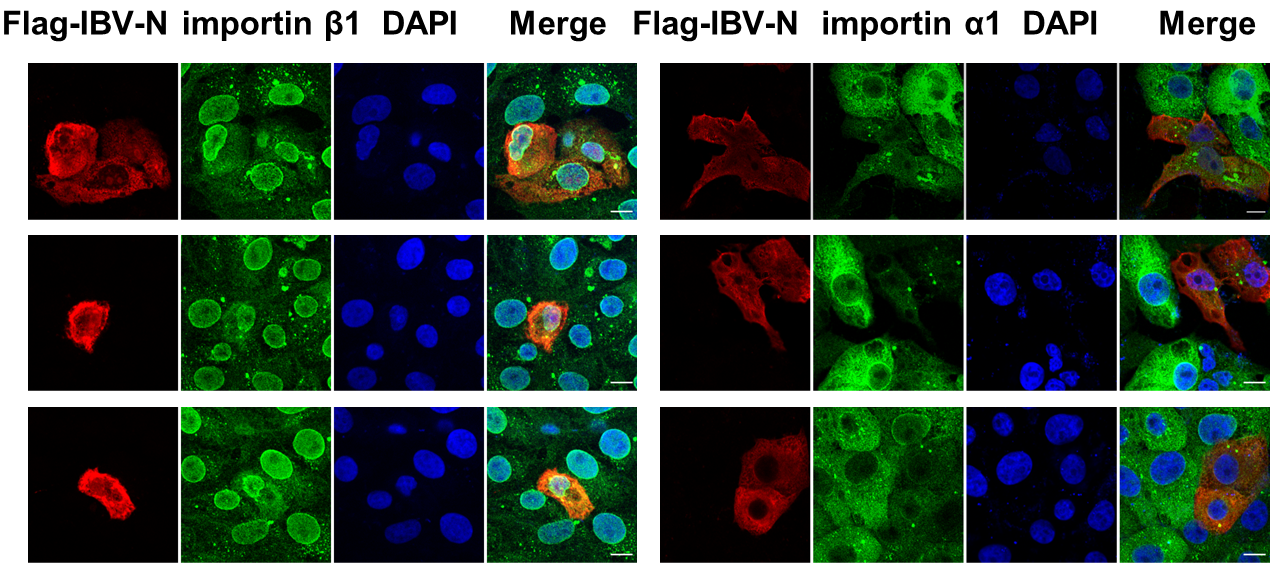

Supplement: S3 Fig — Vero cells were transfected with either the vector PXJ40 or a plasmid encoding IBV N protein. At 24 h post-transfection, cells were harvested and subjected to immunofluorescence analysis. (TIF) [file ppat.1012097.s003.tif]

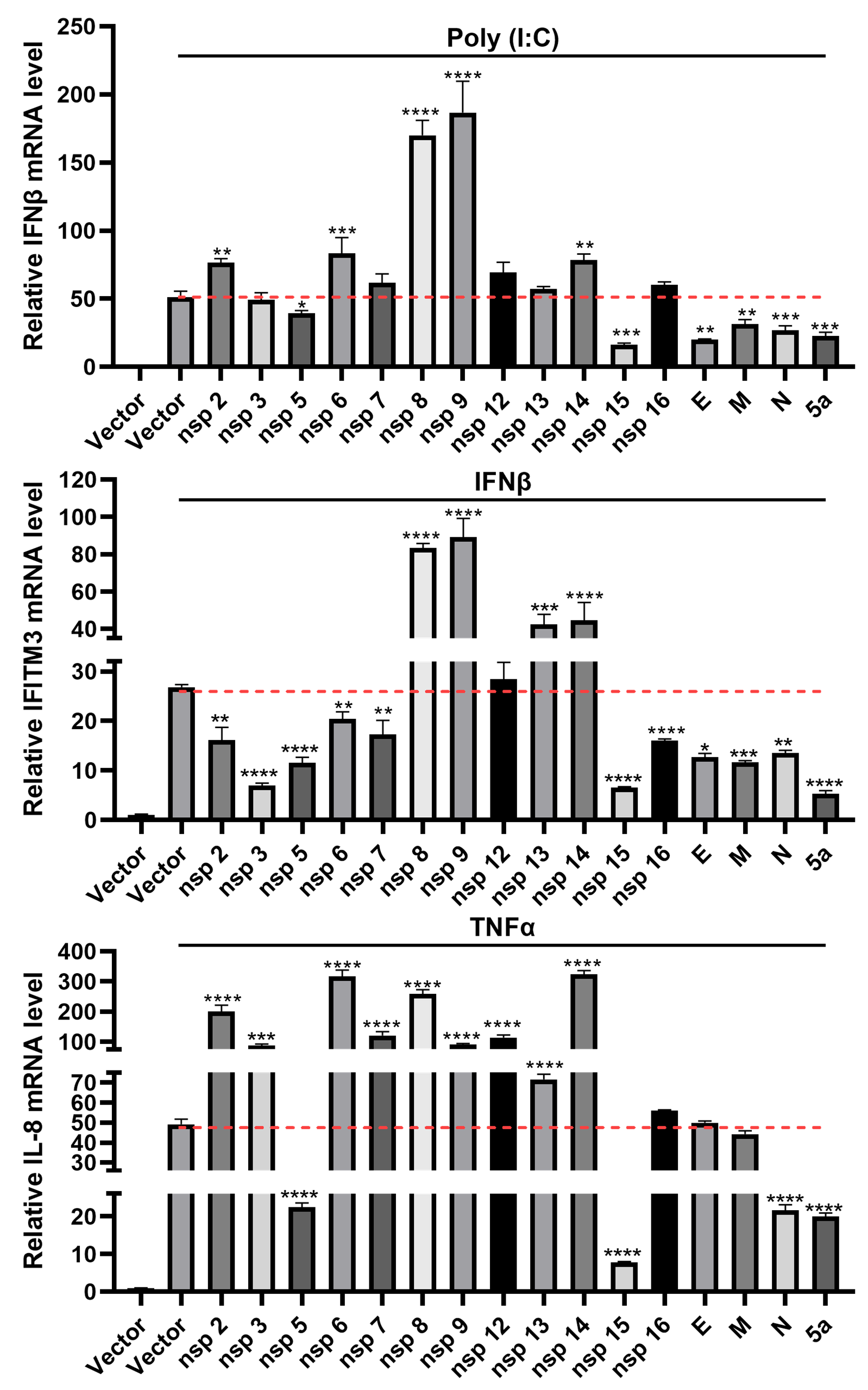

Supplement: S4 Fig — DF-1 cells were transfected with the PXJ40 vector or a plasmid encoding IBV proteins. At 24 h post-transfection, cells were transfected with poly(I:C) or treated with IFNβ or TNFα for 12 h, followed by qRT-PCR analysis. Statistical significance levels are denoted as follows: ns, P > 0.05; *P < 0.05; **P < 0.01; ***P < 0.001; ****P < 0.0001. (TIF) [file ppat.1012097.s004.tif]

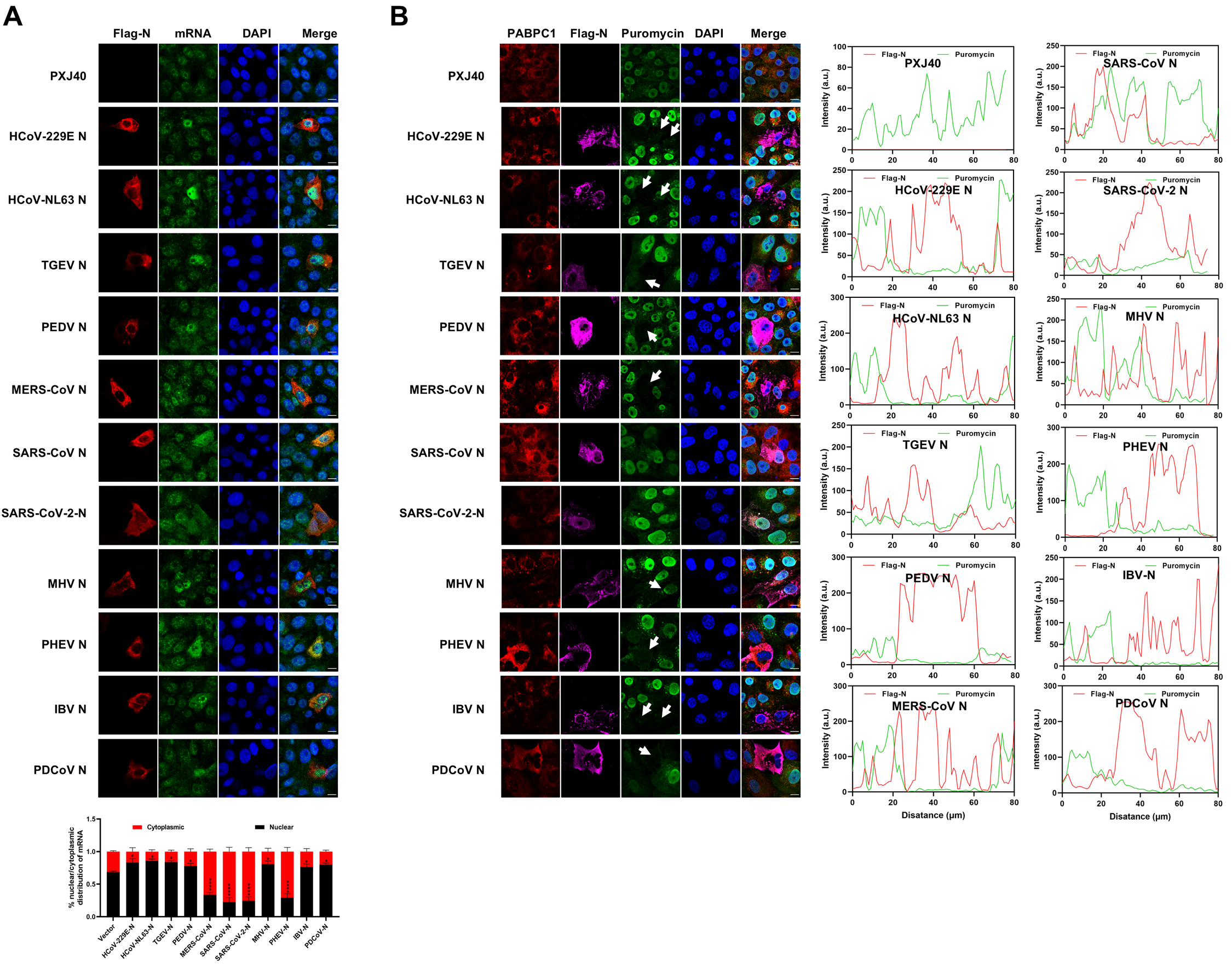

Supplement: S5 Fig — (A) Vero cells were transfected with plasmids encoding Flag-tagged N protein from the indicated coronaviruses or the PXJ40 vector. After 24 h, mRNA was visualized using oligo dT probes (green) via in situ hybridization, followed by indirect immunofluorescence to detect the N protein (red). Nuclei were labeled with DAPI (blue). Representative images from three independent experiments are shown. The fluorescence signals of mRNA in vector transfected cells and N expressing cells from three fields of view were quantified using ImageJ. The intensities of the fluorescence signals in the nucleus (black bars) and the cytoplasm (red bars) are presented as bar graphs. Error bars represent the SD. Statistical significance levels are denoted as follows: *P < 0.05; ****P < 0.0001. (B) Vero cells were transfected with PXJ40 or with a plasmid encoding Flag-tagged N protein from the indicated coronaviruses. After 23 h, puromycin labeling (5 μg/mL) was performed for 1 h. Indirect immunofluorescence was then used to detect PABPC1 (red), N protein (magenta) and puromycin-labeled de novo synthesized peptides (green). Representative images from three independent experiments are shown. The relative fluorescence intensities of the IBV N protein and puromycin were quantified using ImageJ. The relative signal intensities and distributions of N and puromycin-labeled nascent peptides are shown as graph in the right panel. (TIF) [file ppat.1012097.s005.tif]

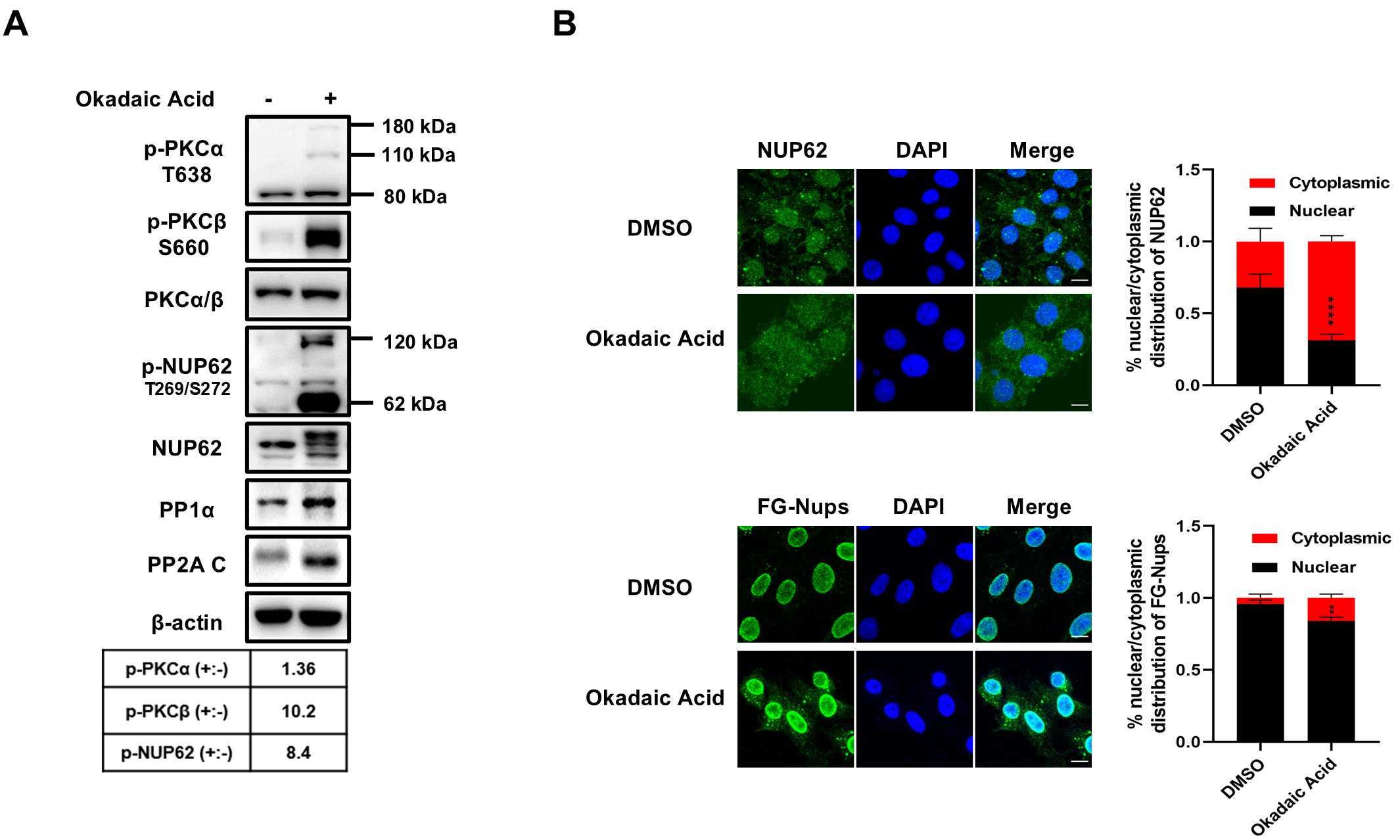

Supplement: S6 Fig — (A) DF-1 cells were treated with either DMSO or okadaic acid (1 μM) for 1 h and subjected to western blot analysis using the indicated antibodies. The intensities of p-PKCα, p-PKCβ, and p-NUP62 bands were normalized to total PKCα/β or NUP62. The ratio of p-PKCα, p-PKCβ, and p-NUP62 in okadaic acid-treated cells to DMSO-treated cells is denoted as p-PKCα (+:-), p-PKCβ (+:-), and p-NUP62 (+:-). (B) Vero cells were treated with either DMSO or okadaic acid (1 μM) for 1 h and subjected to immunostaining. Representative images from three independent experiments are shown, with scale bars indicating 10 μm. The fluorescence signals of Nup62 and FG-Nups in DMSO-treated and okadaic acid-treated cells were quantified from three fields of view using ImageJ. The intensities of the fluorescence signals in the nucleus (black bars) and the cytoplasm (red bars) are presented as bar graphs, with error bars representing the SD. Statistical significance is indicated as **** for P < 0.0001 and ** for P<0.01. (TIF) [file ppat.1012097.s006.tif]

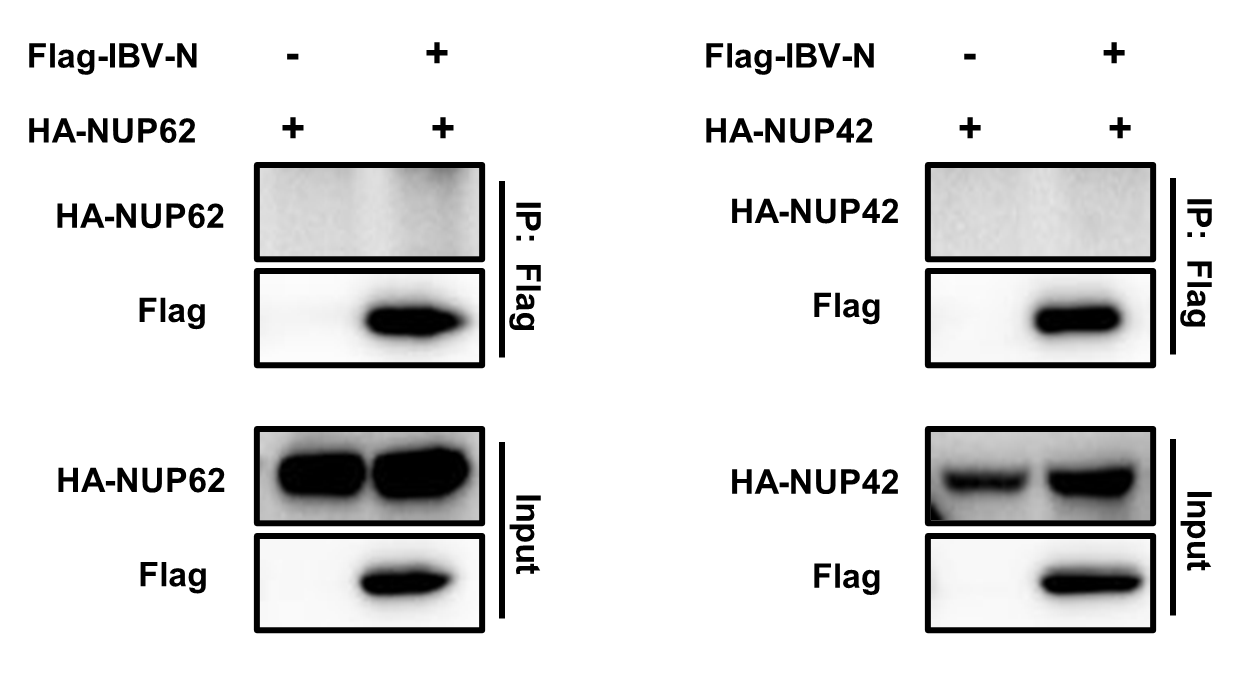

Supplement: S7 Fig — Plasmids encoding HA-tagged NUP62 or HA-tagged NUP42 were co-transfected with Flag-tagged IBV N protein or the PXJ40 control plasmid into HEK-293T cells for 24 h. The cell lysates were collected and subjected to Co-IP using an anti-Flag antibody to isolate the Flag-tagged N protein and any associated proteins. The immunoprecipitated complexes were then analyzed by Western blot analysis to detect the presence of HA-tagged NUP62 or HA-tagged NUP42, as well as the Flag-tagged IBV N protein. (TIF) [file ppat.1012097.s007.tif]
