# Supplementary material for: Coronavirus nucleocapsid protein enhances the binding of p-PKCα to RACK1: Implications for inhibition of nucleocytoplasmic trafficking and suppression of the innate immune response
Source: PLoS Pathog. 2024 Nov 27;20(11):e1012097. doi: 10.1371/journal.ppat.1012097 (PMC11633972; doi:10.1371/journal.ppat.1012097)
Supplement: S1 Table — (DOCX) [file ppat.1012097.s008.docx]

**Table S1 Dilution of primary antibodies and their cross-reactivity with corresponding chicken proteins**

| Antibody name | Application and dilution | Cross-reactivity with  chicken protein |
| --- | --- | --- |
| Anti-IBV-N | Western blot (1:5000)  Immunofluorescence (1:500) | NA  NA |
| Anti-IRF3 | Immunofluorescence (1:100) | Yes |
| Anti-p-IRF3 | Western blot (1:1000) | Yes |
| Anti-p65 | Immunofluorescence (1:100) | Yes |
| Anti-p-p65 | Western blot (1:1000) | Yes |
| Anti-STAT1 | Immunofluorescence (1:100) | No |
| Anti-p-STAT1 | Western blot (1:1000) | Yes |
| Anti-STAT2 | Immunofluorescence (1:100) | Yes |
| Anti-p-STAT2 | Western blot (1:1000) | Yes |
| Anti-IRF9 | Immunofluorescence (1:100) | No |
| Anti-p38 | Immunofluorescence (1:100) | No |
| Anti-p-p38 | Western blot (1:1000) | Yes |
| Anti-NUP62 | Western blot (1:1000)  Immunofluorescence (1:100) | Yes  Yes |
| Anti-p-NUP62 | Western blot (1:1000) | Yes |
| Anti-FG-Nups (mAb414) | Immunofluorescence (1:500) | No |
| Anti-NUP153 | Western blot (1:1000)  Immunofluorescence (1:500) | Yes  Yes |
| Anti-NUP98 | Western blot (1:1000)  Immunofluorescence (1:100) | Yes  Yes |
| Anti-NUP42 (hCG1) | Western blot (1:1000)  Immunofluorescence (1:50) | Yes  No |
| Anti-TPR | Western blot (1:1000)  Immunofluorescence (1:400) | Yes  Yes |
| Anti-Ran | Western blot (1:1000)  Immunofluorescence (1:100) | Yes  Yes |
| Anti-Importin β1 | Western blot (1:1000)  Immunofluorescence (1:100) | Yes  No |
| Anti-Importin α1 | Western blot (1:1000)  Immunofluorescence (1:100) | Yes  No |
| Anti-RACK1 | Western blot (1:1000)  Immunofluorescence (1:100) | Yes  No |
| Anti-PKCα/β | Western blot (1:1000)  Immunofluorescence (1:200) | Yes  Yes |
| Anti-p-PKCα | Western blot (1:1000) | Yes |
| Anti-p-PKCβ | Western blot (1:1000) | Yes |
| Anti-HA | Western blot (1:1000)  Immunofluorescence (1:500) | NA  NA |
| Anti-Flag | Western blot (1:1000)  Immunofluorescence (1:500) | NA  NA |
| Anti-Flag (chicken) | Immunofluorescence (1:1000) | NA |
| Anti-PP1α | Western blot (1:1000) | Yes |
| Anti-PP2A C | Western blot (1:1000) | Yes |
| Anti-PABP1 | Immunofluorescence (1:1000) | Unknown |
| Anti-puromycin | Immunofluorescence (1:1000) | Yes |
| Anti-β-actin | Western blot (1:1000) | Yes |
