# Supplementary material for: Coronavirus nucleocapsid protein enhances the binding of p-PKCα to RACK1: Implications for inhibition of nucleocytoplasmic trafficking and suppression of the innate immune response
Source: PLoS Pathog. 2024 Nov 27;20(11):e1012097. doi: 10.1371/journal.ppat.1012097 (PMC11633972; doi:10.1371/journal.ppat.1012097)
Supplement: S2 Table — (DOCX) [file ppat.1012097.s009.docx]

**Table S2 Primer sequences used for plasmid construction**

|  | **Primer Name** | **Sequence** |
| --- | --- | --- |
| 1 | IBV N Forward | 5'-ATGGACTACAAGGACGACGATGAT-3' |
| 2 | IBV N Reverse | 5'-TAATTACCTTGGGCTCATCATAAA-3' |
| 3 | IBV N ΔNTD Forward | 5'-AAAGTCGGTAACCGTGGTAGGAGTGGAAGATCA-3' |
| 4 | IBV N ΔNTD Reverse | 5'-ACCACGGTTACCGACTTTAGGTGGTTTTGGTCC-3' |
| 5 | IBV N ΔSR Forward | 5'-TGGTAGGGATTCTGGAGATGACCTTATTGCTCG-3' |
| 6 | IBV N ΔSR Reverse | 5'-TCTCCAGAATCCCTACCACGGTTCAGGGGAATG-3' |
| 7 | IBV N ΔCTD Forward | 5'-TCTCGCATTGACGAACCAAAACCAAAGTCACGC-3' |
| 8 | IBV N ΔCTD Reverse | 5'-TGGTTCGTCAATGCGAGAGCCCTTTTTCTGCTG-3' |
| 9 | IBV N ΔNES Forward | 5'-GAGTGACACCCAAAAGGTTTGAATTTACTACTGTGG  TCCC-3' |
| 10 | IBV N ΔNES Reverse | 5'-ACCTTTTGGGTGTCACTCTACTTCCAAAAAGAC-3' |
| 11 | NUP42 Forward | 5'-CGGAATTCGGATGGCCATTTGTCAATTCTTCCTT-3' |
| 12 | NUP42 Reverse | 5'-CGGGGTACCTTAAACATTTAGAAGTTCCAGAGGT-3' |
| 13 | NUP62 Forward | 5'-TCCATGGAGGCCCGAATTCTTATGAGCGGGTTTAATT  TTGGAG-3' |
| 14 | NUP62 Reverse | 5'-GATCCCCGCGGCCGCGGTACCTCAGTCAAAGGTGAT  CCGGAA-3' |
| 15 | RACK1 Forward | 5'-GGTCGACCGAGATCTCTCGAATGACTGAGCAGATGA  CCCTTCG-3' |
| 16 | RACK1 Reverse | 5'-GTCTGGATCCCCGCGGCCGCCTAGCGTGTGCCAATG  GTCA-3' |
| 17 | pCMV-HA Forward | 5'-GCGGCCGCGGGGATCCAG-3' |
| 18 | pCMV-HA Reverse | 5'-TCGAGAGATCTCGGTCGACCG-3' |
| 19 | NLS-EGFP Forward | 5'-TCAGATCTCGAGCTCAAGCTTATGCCAAAAAAGAAG  AGAAAGGTAGGGCCAAAAAAGAAGAGAAAGGTAGGG  GTGAGCAAGGGCGAGG-3' |
| 20 | NLS-EGFP Reverse | 5'-ATGGTGGCGACCGGTGGATCCCGCTTGTACAGCTCG  TCCATGCC-3' |
