# Supplementary material for: Coronavirus nucleocapsid protein enhances the binding of p-PKCα to RACK1: Implications for inhibition of nucleocytoplasmic trafficking and suppression of the innate immune response
Source: PLoS Pathog. 2024 Nov 27;20(11):e1012097. doi: 10.1371/journal.ppat.1012097 (PMC11633972; doi:10.1371/journal.ppat.1012097)
Supplement: S3 Table — (DOCX) [file ppat.1012097.s010.docx]

**Table S3 Primer sequences used for real-time qPCR**

|  | **Primer Name** | **Sequence** |
| --- | --- | --- |
| 1 | ch-IFNβ Forward | 5'-AGCTCTCACCACCACCTTCTC-3' |
| 2 | ch-IFNβ Reverse | 5'-TGGCTGCTTGCTTCTTGTCCTT-3' |
| 3 | ch-IFITM3 Forward | 5'-TGGTGACGGTGGAGACG-3' |
| 4 | ch-IFITM3 Reverse | 5'-GGCAACCAGGGCGATGA-3' |
| 5 | ch-IL8 Forward | 5'-GCAGTTCTGGCTCTCCTCCTGGTTT-3' |
| 6 | ch-IL8 Reverse | 5'-GCTCGGTGTCAGCTTCACATCTTG-3' |
| 7 | ch-actin Forward | 5'-TATTGCTGCGCTCGTTGTTGAC-3' |
| 8 | ch-actin Reverse | 5'-GATACCTCTTTTGCTCTGGGCTTC-3' |
| 9 | hu-IFNβ Forward | 5'-GCTTGGATTCCTACAAAGAAGCA-3' |
| 10 | hu-IFNβ Reverse | 5'-ATAGATGGTCAATGCGGCGTC-3' |
| 11 | hu-IFITM3 Forward | 5'-CTGGGCTTCATAGCATTCGCC-3' |
| 12 | hu-IFITM3 Reverse | 5'-AGATGTTCAGGCACTTGGCGGT-3' |
| 13 | hu-IL8 Forward | 5'-CTTGGTTTCTCCTTTATTTCTA-3' |
| 14 | hu-IL8 Reverse | 5'-GCACAAATATTTGATGCTTAA-3' |
| 15 | hu-actin Forward | 5'-CCAGACATCAGGGTGTGATGG-3' |
| 16 | hu-actin Reverse | 5'-CTCCATATCATCCCAGTTGGTGA-3' |
| 17 | IBV gene 1 Forward | 5'-GTTCTCGCATAAGGTCGGCTA-3' |
| 18 | IBV gene 1 Reverse | 5'-GCTCACTAAACACCACCAGAAC-3' |
| 19 | IBV N Forward | 5'-GAAGAAAACCAGTCCCAGA-3' |
| 20 | IBV N Reverse | 5'-TTACCAGCAACCCACAC-3' |
