# Supplementary material for: Coronavirus nucleocapsid protein enhances the binding of p-PKCα to RACK1: Implications for inhibition of nucleocytoplasmic trafficking and suppression of the innate immune response
Source: PLoS Pathog. 2024 Nov 27;20(11):e1012097. doi: 10.1371/journal.ppat.1012097 (PMC11633972; doi:10.1371/journal.ppat.1012097)
Supplement: Attachment — Submitted filename: Response letter 20240814.docx [file ppat.1012097.s011.docx]

**Shanghai Veterinary Research Institute**

**Chinese Academy of Agricultural Sciences**

Aug 14, 2024

Editors-in-Chief

*Plos Pathogens*

Dear Editor,

I am writing to resubmit our manuscript entitled, “**Coronavirus Nucleocapsid Protein Enhances the binding of p-PKCα to RACK1: Implications for Inhibition of Nucleocytoplasmic Trafficking and Suppression of the Innate Immune Response**”, for consideration of publication in *PLOS Pathogens*. We are grateful for the opportunity to revise our work in response to the constructive feedback provided by you and the reviewers. We have carefully considered all the comments and suggestions made by reviewers and have made the necessary revisions to improve the manuscript. Below are the responses to editors’ and reviewers’ comments and suggestion.

Dear Authors - Please kindly consider the Reviewers comments, addressing all of their concerns. Particularly pay attention to careful quantification and statistics for all data as well as the additional controls recommended by Reviewer 2. While the conclusions drawn may be valid it is not possible to draw these conclusions from a single representative image. Quantification of multiple cells must be included with relevant statistics and carefully worded methods into the number of samples/images/cells analyzed etc. Additionally, some insight should be added on how this relates to real-world physiological animal infections and the larger impact of the work, also with relevance to existing literature. We find the conclusions interesting to the field, nonetheless, and look forward to seeing a revised manuscript.

**Response:** Dear editor, thank you for your constructive feedback and for highlighting the need for careful quantification, statistics, and additional controls. We appreciate your suggestions and have made the following revisions to address the concerns:

1. **Quantification and Statistics:** We have included comprehensive quantification of three fields of view per condition across all immunostaining experiments, with statistical analyses. The methods section has been updated to clearly outline the three fields of view analyzed to ensure reproducibility and robustness of the data.
2. **Additional experiments:** We have indeed assessed the phosphorylation levels of IRF3, STAT1, p65, and p38 following IBV infection in Fig 1. To complement the data presented in Fig 4D, we conducted additional experiments to identify IBV proteins that counteract the host innate immune response (S4 Fig). Additionally, we performed FISH and puromycin labeling experiments to demonstrate that N protein expression results in mRNA nuclear retention and reduced protein translation levels (S5 Fig). We have also added RT-PCR and plaque assay data to validate the impact of Enzastaurin and down-regulation of PKC and RACK1 on viral replication (Fig 9E-9F, Fig 10B-10C).
3. **Impact of the Work:** We have elaborated on the significance of our work in the broader context of coronavirus pathogenesis and potential therapeutic interventions.
4. **Relation to Real-World Physiological Infections:** We have expanded the discussion to provide insights into how our findings relate to real-world physiological animal infections.

**Part I - Summary**
Please use this section to discuss strengths/weaknesses of study, novelty/significance, general execution and scholarship.

**Reviewer #1:** Overall, this manuscript presents a comprehensive investigation into the mechanisms by which the infectious bronchitis virus (IBV) interferes with the host's immune response through manipulation of nucleocytoplasmic trafficking. This study represents the first comprehensive report unveiling that coronaviruses harbor conserved mechanisms, orchestrated by the nucleocapsid protein, to manipulate nucleocytoplasmic trafficking and suppression of nuclear entry of multiple transcription factors. Additionally, it elucidates the pivotal role of the N-RACK1-PKC axis in Nup62 phosphorylation and disassembly of nuclear pore complex (NPC) integrity. Furthermore, this study provides direct evidence of the correlation between Nup phosphorylation and the NPC disassembly. It highlights the impact of coronavirus infection on NPC integrity and functionality, shedding light on novel mechanisms by which pan coronaviruses subverts host innate immune defense.

**Response:** Thank you for your thorough review and positive feedback on our manuscript. We appreciate your recognition of the comprehensive nature of our study and the insights it provides into the mechanisms by which IBV interferes with the host immune response. We are pleased that you found our investigation into the nucleocapsid protein's role in manipulating nucleocytoplasmic trafficking and the suppression of nuclear entry of transcription factors to be valuable. In response to your feedback, we have addressed your concerns in our revised manuscript. We hope these revisions address your comments and enhance the manuscript.

**Reviewer #2:** In this manuscript submitted to Plos Pathogens, the authors aimed to address the impact of coronavirus infection on the nucleocytoplasmic trafficking of host cells, focusing mainly on infection by the gamma-coronavirus, Infectious Bronchitis Coronavirus; IBV). Previous studies already addressed how other coronaviruses (e.g., SARS-CoV and SARS-CoV-2) interfered with the nuclear transport system to suppress innate immunity (e.g., Frieman et al. J Virol. 2007 doi: 10.1128/JVI.01012-07; Miorin L et al. PNAS 2020 doi: 10.1073/pnas.2016650117 and Hall et al. PLoS Pathog. 2022 doi: 10.1371/journal.ppat.1010349). This inhibition involves in particular ORF6, which is absent in other coronaviruses, yet previous studies showed that IBV also inhibited IFNβ-mediated nuclear translocation of STAT1 (e.g., Kint et al. J Virol. 2015 doi: 10.1128/JVI.01057-15).

Here, the authors showed that IBV-induced cytoplasmic dispersion of several FG-Nups blocked the nuclear ingress of various transcription factors (i.e., IRF3, STAT1, STAT2 and, in part, p65 and p38 MAPK), and consequently the transcription of antiviral and pro-inflammatory genes. They further revealed that the IBV N protein contribute to this disturbance of the host nucleocytoplasmic trafficking by enhancing the association of p-PKCα with scaffold protein RACK1, leading to relocation of the p-PKCα-RACK1 complex to cytoplasm. Next, they propose that this event implied the phosphorylation and cytoplasmic dispersion of NUP62. Finally, they showed that the disruption of NPC integrity and interference with nuclear translocation of transcription factors by the N protein is conserved for different coronaviruses.

The topic and issues addressed are of great interest, as the modulation of nuclear translocation and antiviral responses was previously focused on the inhibition by ORF6, which is not conserved across different coronaviruses.
Despite the deep molecular analyses of this manuscript, for virtually all the conclusions on the perturbation of subcellular localization of regulators, only single representative imagings are presented. Therefore, the authors should include a quantification of these phenotypes. This main limitation along with a few others critic required additional experiments to better support the authors' conclusions, as suggested in these specific points.

**Response:** Thank you for your detailed review and insightful comments on our manuscript. We appreciate your acknowledgment of the importance of our study in the context of nucleocytoplasmic trafficking and the impact of IBV on host immune responses. We understand the concerns raised regarding the need for quantification of the observed phenotypes and additional experiments to support our conclusions. In response to your feedback, we have made the following revisions:

1. **Quantification of Phenotypes:** We have conducted quantitative analyses of the nuclear and cytoplasmic distribution ratios of the transcription factors (IRF3, STAT1, STAT2, p65, and p38 MAPK) and nuclear pore complex components (NUP62, NUP153, NUP42, and TPR) based on immunofluorescence results (each quantification includes cells in three fields of view). These quantifications are presented in the revised figures and include relevant statistics to support our conclusions robustly.
2. **Additional Experiments:** We have indeed assessed the phosphorylation levels of IRF3, STAT1, p65, and p38 following IBV infection in Fig 1. To complement the data presented in Fig 4D, we conducted additional experiments to identify IBV proteins that counteract the host innate immune response (S4 Fig). We have also added RT-PCR and plaque assay data to validate the impact of Enzastaurin and down-regulation of PKC and RACK1 on viral replication (Fig 9E-9F and Fig 10B-10-C).
3. **Introduction of Prior Studies:** We have incorporated relevant background information on how other coronaviruses, such as SARS-CoV ORF6, SARS-CoV-2 ORF6, MERS-CoV 4b, affect nuclear transport and innate immunity.
4. **Broader Implications:** We have expanded our discussion to relate our findings to real-world physiological infections and their broader impact. This includes insights into how our study contributes to understanding coronavirus pathogenesis and potential therapeutic interventions.

We hope these revisions address your concerns and strengthen the manuscript.

**Reviewer #3:** The study uncovers a novel mechanism by which the coronavirus N protein interferes with the nuclear transport system, specifically through the interaction with RACK1 and p-PKCα, leading to the phosphorylation and cytoplasmic dispersion of NUP62. The research includes extensive immunofluorescence and biochemical analyses, providing robust evidence for the inhibitory effects of the IBV N protein on nuclear translocation of key transcription factors like IRF3, STAT1, STAT2, and p65. The findings demonstrate that the mechanism is conserved among N proteins from multiple coronaviruses, indicating a potentially universal strategy employed by these viruses to evade host immune responses. By elucidating a previously unknown mechanism of immune evasion employed by coronaviruses, the study provides critical insights that could inform the development of new therapeutic strategies targeting the NPC and related pathways to counteract coronavirus infections.

**Response:** Thank you for your positive and insightful feedback on our manuscript. We appreciate your recognition of the comprehensive investigation and the novel findings we have presented.

**Part II – Major Issues: Key Experiments Required for Acceptance**

**Reviewer #1:** Here are some review comments. Addressing these points would help strengthen the manuscript.

1. Improvements are needed in the presentation of immunostaining figures, as the target cells appear too small for clear visualization. Enlarging the target cells would enhance visibility.

**Response:** Thank you for highlighting the issue with the presentation of the immunostaining figures. We understand the importance of clear visualization for accurate interpretation. We have addressed this concern by quantifying the fluorescence signals in the target cells of three fields of view using Image J software. To provide a clearer representation of our data, we have quantified the nuclear-to-cytoplasmic signal ratio of target proteins and presented this information as bar graphs in all the immunostaining figures. This method ensures that the data is presented in a way that enhances visibility and interpretability, regardless of the size of the target cells in the images.

2. Is the inhibition of nuclear entry by the N protein applicable to all types of nuclear localization signals (NLS)? Including reporter plasmids with NLS would indeed be beneficial for investigating the specificity of the inhibition of nuclear entry by the N protein.

**Response:** Thank you for your insightful comment. Our findings demonstrate that the N protein indeed inhibits the nuclear import of various transcription factors containing various NLS sequences. For example, The NLS for IRF3 is RTQKRLR, the NLS for STAT1 is KGKKTK (407-412 aa), the NLS for STAT2 is KRKRK (733-737 aa), the NLS for IRF9 is KRKR, and the NLS sequence for p65 is RKRR. As shown in Figure 5, the N protein inhibits the nuclear import mediated by all these NLS sequences, indicating that the inhibition is not specific to a particular type of NLS but rather is a general phenomenon affecting various NLS-directed nuclear imports. To investigate this further, we constructed a GFP-GFP tandem plasmid with the SV40 large T antigen NLS sequence (PKKKRKV) and co-transfected it with the N protein plasmid. Immunofluorescence analysis confirmed that N proteins from different coronaviruses inhibit the PKKKRKV-directed nuclear import of GFP. We have included this new data in Figure 5.

Although p38 MAPK does not contain classical NLS or nuclear export signals (NES), it rapidly and robustly translocates to the nucleus and is exported back to the cytoplasm within minutes to hours. This translocation does not seem to occur through interaction with classical importins α/β or via passive diffusion. Instead, the nuclear shuttling of p38 is mediated by importins 3, 7, and 9. Upon cellular stimulation, p38 is released from its cytoplasmic anchoring proteins and interacts with either importin 7 or importin 9, each forming a complex with importin 3. The resulting trimeric complexes (Imp7/Imp3/p38 or Imp9/Imp3/p38) are then shuttled to the nuclear pore complex (NPC). At the NPC, importin 3 remains, while importin 7 or importin 9 escorts the p38 into the nucleus. The importin 7 binding site of p38 is located in the N-terminus of the kinase and consists of at least nine amino acid residues: PERYQNLSP. Deletion of this region or substitution of its residues with alanine prevents the interaction with importin 7. The inhibition of p38’s nuclear translocation by the coronavirus N protein further demonstrates that the N protein targets the nuclear pore complex to interfere with nuclear trafficking.

3. Has the author investigated whether the N protein inhibits the nuclear export of host mRNA and subsequent protein translation?

**Response:** Yes, we have investigated the effect of the N protein on the nuclear export of host mRNA and subsequent protein translation. Our experiments demonstrate that most coronaviruses N protein inhibits the export of mRNA from the nucleus to the cytoplasm, which in turn affects protein translation. We have included the relevant experimental data and analysis in the supplementary material as S5 Fig. This figure provides detailed results supporting the inhibitory effect of the N protein on mRNA nuclear export and translation processes.

4. The specific roles of PKCα and PKCβ in phosphorylating Nup62 were not addressed in this study. Is it feasible to individually knock down PKCα or PKCβ using specific siRNAs to investigate their respective contributions to Nup62 phosphorylation?

**Response:** Thank you for raising this important point. We attempted to elucidate the individual contributions of PKCα and PKCβ to the phosphorylation of Nup62 by designing and using specific siRNAs targeting each isoform separately. However, during our experiments, we encountered a significant challenge: the siRNAs designed to target PKCα and PKCβ individually resulted in the simultaneous knockdown of both isoforms. Despite several redesigns and optimizations of the siRNA sequences, we were unable to achieve selective knockdown of either PKCα or PKCβ. As a result, we were unable to definitively determine which isoform is primarily responsible for the phosphorylation of NUP62.

Nonetheless, our immunoprecipitation experiments provide some insight into the potential roles of these kinases. The results indicate that the N protein recruits a greater amount of PKCα to RACK1, suggesting that the N protein facilitates the translocation of the PKCα-RACK1 complex within the cell. This translocation may enhance the phosphorylation of NUP62. However, it is important to note that viral infection has been shown to increase the phosphorylation levels of PKCβ, which suggests that PKCβ may also contribute to the phosphorylation of NUP62.

In summary, while our results suggest a role for PKCα in the phosphorylation of NUP62, we cannot exclude the possibility that PKCβ also plays a role in this process. Further studies using alternative methods to selectively inhibit PKCα and PKCβ or the use of more advanced gene editing techniques might be necessary to fully elucidate the individual contributions of these kinases.

5. Based on this study, deleting the nuclear export signal (NES) of the N protein could affect its subcellular localization and function. Is it feasible to rescue a recombinant mutant virus by deleting the NES from the N protein? It is understood that in some cases, deleting essential viral elements like the NES may severely impair viral replication or assembly, making it difficult to rescue viable mutant viruses.

**Response:** Thank you for your insightful comment. As you pointed out, constructing a recombinant IBV lacking the NES (∆NES) of the N protein is critical to verify and understand the role of the N protein in regulating nuclear-cytoplasmic trafficking in vivo. We have made several attempts to generate the rIBV-N-∆NES recombinant virus using reverse genetic technology. Unfortunately, these efforts have not been successful, and we have been unable to rescue a viable rIBV-N-∆NES virus.

Our findings suggest that the nuclear export of the N protein is a vital process for IBV replication, and the deletion of the NES appears to result in a lethal mutation. This suggests that the NES is essential for the proper replication and assembly of the virus, and its removal impairs these processes significantly. The inability to rescue the mutant virus further supports the hypothesis that the N protein’s nuclear export function is crucial for the virus’s life cycle.

We have discussed these findings and their implications in greater detail in the Discussion section of our manuscript. This section elaborates on the potential mechanisms by which the loss of the NES may disrupt viral replication and assembly, leading to a non-viable virus. We believe that further investigations using alternative strategies or compensatory mutations might be necessary to explore this area more thoroughly.

**Reviewer #2:**

1. The imaging panels of Figure 1 clearly showed that IBV-mediated inhibition of nuclear translocation IRF3, STAT1 and STAT2, yet this is not readily observed for p65 and p38, as partly localized in the cytosol. The authors should thus reinforce their conclusions by a quantification of the cytosol versus nuclear subcellular localization of the different regulators for the tested conditions and presented as means for independent experiments. For example, this can be performed by quantifying their relative levels of colocalization with DAPI on the imaging analysis and/or by western blot analyses upon biochemical separation of nuclear and cytoplasm fractions.

**Response:** Thank you for your valuable feedback and suggestions. We acknowledge the challenge of accurately observing the inhibition of transcription factor nuclear entry due to viral infection, especially in the early stages when only a small fraction of cells are infected, and most cells continue to exhibit nuclear entry of transcription factors under the influence of stimulatory factors. This situation makes it difficult to detect changes in the nuclear-cytoplasmic distribution of transcription factors using traditional biochemical fractionation and Western blot analysis.

To address this issue and reinforce our conclusions, we have conducted a quantitative analysis of the nuclear and cytoplasmic distribution ratios of IRF3, STAT1, STAT2, p65, and p38 using immunofluorescence. The results of our quantitative analysis are now included in the revised figures of the manuscript. We believe that this additional data enhances the clarity and robustness of our conclusions regarding the impact of IBV on the nuclear translocation of these critical transcription factors.

2. Similar quantifications should be performed for NUP62, NUP153, NUP42, and TPR redistribution to the cytoplasm upon IBV infection shown in Figure 2 (especially since this observation is transient/vanish at late time-points for some regulators), as well as upon expression of the individual viral proteins (Figure 3, 4, 5, 8 and 12) and for Figure 7C and 9B-C.

**Response:** Thank you for your suggestion. We have conducted a comprehensive quantitative analysis of the nuclear and cytoplasmic distribution ratios of NUP62, NUP153, NUP42, and TPR based on the immunofluorescence results. This analysis was performed across various conditions, including:

- Redistribution to the cytoplasm upon IBV infection as shown in Figure 2.
- Redistribution upon expression of individual viral proteins as depicted in Figures 3, 4, 5, 8, and 12.
- Redistribution of shown in Figures 7C and 9B-C.

The results of these analyses are now included in the revised figures of the manuscript. We believe this additional quantitative data enhances the robustness and clarity of our findings regarding the redistribution of NUP62, NUP153, NUP42, and TPR under various experimental conditions.

3. In Figure 4D and 5E, to better conclude on the preferential regulation by N protein, the author should include similar RT-qPCR analyses of antiviral/pro-inflammatory responses upon expression of **other viral proteins**, as a comparison.

**Response:** Thank you for your insightful suggestion. To provide a more comprehensive understanding of the preferential regulation by the N protein, we have performed additional RT-qPCR analyses to evaluate the effects of other IBV-encoded proteins on antiviral and pro-inflammatory gene expression. These analyses were conducted to compare their inhibitory effects with those of the N protein.

The results of these analyses are included in the revised manuscript as S4 Fig. Our findings indicate that, in addition to the N protein, other viral proteins also exhibit inhibitory effects on antiviral gene expression. Notably, the nonstructural proteins nsp5, nsp15, and accessory protein 5a demonstrated significant inhibitory effects on both the interferon signaling pathway and the inflammatory response signaling pathway. Among these proteins, nsp15 showed the most pronounced effect, highlighting the virus’s strategy of utilizing multiple proteins to evade the host's innate immune response.

This study specifically focuses on elucidating the mechanism by which the N protein inhibits the host's innate immune response by interfering with the nuclear transport system. The data presented in S4 Fig underscores the multifaceted approach employed by IBV to modulate the host immune response. We plan to further investigate the mechanisms by which nsp5, nsp15, and 5a inhibit the innate immune response in future research.

4. In Figure 9A, the change of phosphorylation levels of PKC and NUP62 are very limited in Vero cells as compared to DF1 cells. The authors should comment on this difference.

**Response:** Thank you for pointing out this observation. We have performed the experiment multiple times, and the results consistently show that phosphorylation levels of PKC and NUP62 are more pronounced in DF-1 cells compared to Vero cells, as illustrated in Figures 9A and 2C. Despite the more limited changes observed in Vero cells, phosphorylation of NUP62 remains evident, as shown in Figure 2C.

The underlying mechanisms responsible for the differences in phosphorylation levels between these cell lines are not fully understood at this time. We have included a discussion of these differences and their potential implications in the revised manuscript. This section addresses the observed variability and emphasizes the need for further research to elucidate the factors contributing to the differential phosphorylation of PKC and NUP62 in different cell lines.

5. The impact of Enzastaurin inhibitor and down-regulation of PKC or RACK on viral replication is only revealed by analysis of the level of N protein in Figure 9D-E and 10A. Owing to the importance of these conclusions, the results should be confirmed by a different quantitative approach to demonstrate that the impact is on viral replication per se rather than the stability/expression levels of only the intracellular N protein.

**Response:** Thank you for highlighting the need for additional confirmation of our results. To address this concern, we conducted further experiments to assess the impact of the Enzastaurin inhibitor and the down-regulation of PKC or RACK on viral replication more comprehensively. We measured viral genome replication and transcription using RT-PCR. This analysis showed a significant reduction in viral genome replication in cells treated with Enzastaurin or subjected to PKC or RACK down-regulation. We also evaluated the release of infectious virus particles into the culture medium by performing plaque assays. The results demonstrated a clear reduction in the virus titer in Enzastaurin-treated or PKC/RACK-knockdown cells. These additional data confirm that the observed effects are indeed on viral replication, rather than being solely related to changes in the intracellular levels of the N protein. We have included these results in the revised manuscript and updated the corresponding figures to reflect these findings. We believe that these additional experiments provide robust confirmation of the impact of Enzastaurin and PKC/RACK down-regulation on viral replication.

Plaque assay was performed to determine the number of virus particles released into the cell culture supernatant from Vero cells treated with Enzastaurin inhibitor versus DMSO control, or from cells transfected with siRACK1, siPKCα/β or non-target control siRNA (NC). The results are presented as the number of plaques formed.

6. In Figure 9F and 10B-C, the diminished impact of IBV infection on the antiviral response upon siRNA against PKC and RACK can result - at least in part - from an indirect consequence of decreased viral replication and/or stability/expression levels of N protein in down-regulated cells (as mentioned in point 5) and in accordance with results of Figure 11F and 12F upon individual expression of N protein. This question should be addressed.

**Response:** Thank you for raising this important point. We addressed this concern by investigating whether the observed restoration of antiviral gene expression upon siRNA-mediated knockdown of PKC and RACK is a direct effect or an indirect result of decreased viral replication or changes in N protein expression levels. Fig 11F demonstrates that the IBV N protein indeed inhibits the expression of antiviral genes. Importantly, knockdown of RACK1 or PKCα/β restores the expression of these antiviral genes, indicating that the inhibitory effect of the N protein is dependent on PKC and RACK1. Additionally, Figure 11E shows that RACK1 knockdown does not affect the expression levels of the transfected N protein, confirming that the restoration of antiviral gene expression is due to the depletion of RACK1, not a reduction in N protein levels. Our goal was to confirm that the N protein’s ability to inhibit antiviral gene expression relies on PKC and RACK1. The data presented in Figure 11F address the concerns raised regarding Fig 9F and 10B-C, showing that the observed effects are directly related to the role of PKC and RACK1 in mediating the N protein’s function, rather than being an artifact of changes in viral replication.

**Reviewer #3:**

1. The study primarily relies on in vitro models, such as Vero and DF-1 cells, which might not fully replicate the complexity of an in *vivo* system.

**Response**: Thank you for your comment. We acknowledge that in vitro models, such as Vero and DF-1 cells, may not fully replicate the complexity of in vivo systems. However, several factors limit our ability to perform similar studies in vivo:

(1) **Physical and Chemical Stimulation Challenges:** Conducting studies on nuclear entry of transcription factors and transfecting the N protein in animals involves significant technical challenges. Physical or chemical stimulation-related studies in animals are difficult to execute effectively.
(2) **Antibody Availability:** There is a lack of commercial antibodies for various chicken transcription factors and Nups. Additionally, human protein antibodies may have poor cross-reactivity with chicken proteins, making in vivo studies problematic.
(3) **Resolution Limitations:** Immunohistochemistry, while useful, does not achieve the same resolution as confocal microscopy used in immunofluorescence experiments. The lower resolution of immunohistochemistry can limit the ability to clearly visualize and analyze subcellular localization and interactions. Even if viral infection were used to examine the nuclear entry of transcription factors and the distribution of NUPs in chicken tissues, immunohistochemistry may not provide sufficiently clear results to draw definitive conclusions.
(4) **Cytokine Detection:** While RT-PCR or ELISA can detect cytokine expression in infected animals, the levels of cytokine expression may not provide sufficient insight into the underlying mechanisms in chickens.

Given these limitations, mechanistic studies related to the nuclear entry of transcription factors and Nups redistribution upon virus infection or viral protein expression are currently best performed using cell-based models. We are working towards developing additional approaches that may address these limitations in future research.

2. The phosphorylation of IRF3, STAT1, p65 and p38 should be detected in figure 1, which may help to clarify the role of NPC in the suppression of IFNβ, IFITM3, and IL-8.

**Response:** Thank you for your suggestion. We have indeed assessed the phosphorylation levels of IRF3, STAT1, p65, and p38 following IBV infection. Our data show that while IBV infection induces phosphorylation and activation of IRF3, it does not facilitate its nuclear entry. Specifically, IBV infection inhibits the nuclear translocation of IRF3 despite poly(I:C)-stimulated IRF3 phosphorylation. This suggests that the virus targets IRF3 at the level of nuclear translocation, rather than affecting its phosphorylation. IBV infection does not enhance STAT1 and STAT2 phosphorylation. In fact, it significantly inhibits IFN-β-induced STAT1 phosphorylation at later stages of infection. Immunofluorescence results indicate that IBV not only prevents the nuclear import of STAT1 and STAT2 but also disrupts STAT1 phosphorylation in the upstream signaling pathway. These observations highlight that IBV interferes with both the phosphorylation and nuclear translocation of these transcription factors. At later stages of infection, IBV shows a slight increase in the phosphorylation of p65 and p38, which correlates with the inflammatory response. Immunofluorescence also indicates that the inhibition of p65 nuclear translocation by IBV is less pronounced, with some p65 still entering the nucleus. This suggests that IBV's regulation of the nuclear transport system is complex and dynamic, with varying effects on different transcription factors.

We have updated Fig 1 to include data on the phosphorylation of these factors and have elaborated on these findings in the revised manuscript to provide a clearer understanding of how IBV affects NPC function and the suppression of antiviral responses.

**Part III – Minor Issues:** Editorial and Data Presentation Modifications
Please use this section for editorial suggestions as well as relatively minor modifications of existing data that would enhance clarity.

**Reviewer #1: Minor comments:**

1. Background on coronaviruses in introduction section: The section detailing coronaviruses' genomic structure and pathogenicity provides essential context for understanding their impact on host cells and the immune response. However, it might be beneficial to briefly mention the zoonotic origins of some coronaviruses (e.g., SARS-CoV and SARS-CoV-2) to highlight their potential for cross-species transmission.

**Response:** Thank you for your suggestion. We have revised the introduction to include a brief discussion on the zoonotic origins of coronaviruses. The updated section now highlights the potential for cross-species transmission, specifically mentioning SARS-CoV, MERS-CoV, and SARS-CoV-2, to provide a more comprehensive context regarding the impact of these viruses on both animal and human populations. The relevant content has been added to lines 149-156 of the introduction.

2. While the manuscript discusses the findings in the context of coronavirus infection, it could benefit from providing more background information on the significance of nucleocytoplasmic trafficking in antiviral immune responses. Offering a brief overview of previous studies or theories in this area would help readers better understand the significance of the current findings.

**Response:** Thank you for your valuable feedback. We have expanded the introduction to include a brief overview of the significance of nucleocytoplasmic trafficking in antiviral immune responses. This addition provides context by summarizing relevant previous studies and theories, which helps to elucidate the importance of our findings in relation to nucleocytoplasmic trafficking. The revised content can be found in lines 110-125 of the introduction section.

3. The transition from discussing general aspects of nucleocytoplasmic trafficking and coronavirus biology to introducing the specific objectives of the study could be smoother. Providing a brief overview or roadmap of the study's aims and methodologies would help orient the reader and set clear expectations for the subsequent sections.

**Response:** Thank you for the constructive suggestion. We have improved the transition by including a brief overview and roadmap of the study's aims and methodologies in the introduction section. This addition helps to bridge the discussion of general concepts with the specific objectives of our study, providing a clearer orientation and setting expectations for the subsequent sections.

4. The manuscript provides detailed experimental results, but there could be further discussion on the implications of these findings. For instance, what are the broader implications of IBV's interference with nucleocytoplasmic trafficking for our understanding of coronavirus pathogenesis and potential therapeutic interventions? Discussing these points would enrich the interpretation of the data.

**Response:** Thank you for your insightful feedback. We have expanded the discussion to include an analysis of the broader implications of IBV’s interference with nucleocytoplasmic trafficking. This addition addresses how our findings contribute to a deeper understanding of coronavirus pathogenesis and explores potential therapeutic interventions.

5. Including graphical representations of key findings, such as graphs presenting quantitative data, could enhance the manuscript's visual appeal and aid in conveying complex concepts more effectively.

**Response:** Thank you for your suggestion. In response, we have incorporated graphical representations of key findings to enhance the visual appeal and clarity of the manuscript. We conducted quantitative analyses of the nuclear and cytoplasmic distribution ratios for transcription factors, NUPs, importins, RACK1, and PKCα/β based on immunofluorescence results. These analyses are now presented in the revised figures of the article.

6. Exploring the size shift of Nup42 in future study could offer intriguing insights into its role in maintaining the integrity of the nuclear pore complex (NPC).

**Response:** Thank you for your valuable suggestion. We have included a discussion on the potential exploration of Nup42 size shift in future studies to gain further insights into its role in maintaining the integrity of the nuclear pore complex (NPC).

**Reviewer #2:**

1- In Figure 9A, the legend is completed: (+) is missing at 18 h.p.i..

**Response:** Thank you for pointing this out. We have updated Fig 9A to include the missing (+) symbol at 18 h.p.i. The revised legend now accurately reflects the experimental conditions.

2- Previously known function of the N protein in coronavirus life cycle and its impact on host cells should be introduced, especially in regards to its known ability to disrupt of host cell division (e.g., as reviewed by Su et al., Front Vet Sci. 2020 doi: 10.3389/fvets.2020.586826).

**Response:** Thank you for the suggestion. We have included a discussion on the previously known functions of the N protein, particularly its role in disrupting host cell division, as highlighted in the review by Su et al. (Front Vet Sci. 2020). This additional information can be found in the revised discussion section.

Reviewer #3: NO
