# Supplementary material for: Coronavirus nucleocapsid protein enhances the binding of p-PKCα to RACK1: Implications for inhibition of nucleocytoplasmic trafficking and suppression of the innate immune response
Source: PLoS Pathog. 2024 Nov 27;20(11):e1012097. doi: 10.1371/journal.ppat.1012097 (PMC11633972; doi:10.1371/journal.ppat.1012097)
Supplement: Attachment — Submitted filename: Response to reviewers 20241109.doc [file ppat.1012097.s012.doc]

**Shanghai Veterinary Research Institute**

**Chinese Academy of Agricultural Sciences**

Nov 9, 2024

Editors-in-Chief

*Plos Pathogens*

Dear Editor,

I am writing to resubmit our manuscript entitled, “**Coronavirus Nucleocapsid Protein Enhances the binding of p-PKCα to RACK1: Implications for Inhibition of Nucleocytoplasmic Trafficking and Suppression of the Innate Immune Response**”, for consideration of publication in *PLOS Pathogens*. We are grateful for the opportunity to revise our work in response to the constructive feedback provided by you and the reviewers. We have carefully considered all the comments and suggestions made by reviewers and have made the necessary revisions to improve the manuscript. Below are the responses to editors’ and reviewers’ comments and suggestion.

Additionally, we have uploaded the raw data to the following database. As the URL upload in the article submission system was consistently unsuccessful, we share the link here for access. https://zenodo.org/records/14067323?preview=1&token=eyJhbGciOiJIUzUxMiJ9.eyJpZCI6Ijk3NGY4Y2IyLWE0M2ItNDI2NS1iOGVmLTVmNGRmZmZkM2Y4MyIsImRhdGEiOnt9LCJyYW5kb20iOiIzZGE2NmEwNjlhOTI4OTIxOGZjYjg1NzNmNzA1NzVlYyJ9.AmQFCLb772-Z8LDAGUVK6lIVdt0wNzP7SYGx2Df52FXjeNkYV4TQxFst2-MmuosNPj4V5AOd8Bdlfx6H9eNDDQ

Your sincerely,

Ying Liao on behalf of the authors

Shanghai Veterinary Research Institute, Chinese Academy of Agricultural Sciences, China

Corresponding author:

Ying Liao, Phone: +86-21-34680291, Fax: +86-21-54081818,

Email: liaoying@ shvri.ac.cn

Thank you for submitting your manuscript to PLOS Pathogens. After careful consideration, we feel that it has merit but does not fully meet PLOS Pathogens's publication criteria as it currently stands. Therefore, we invite you to submit a revised version of the manuscript that addresses the points raised during the review process.

Please submit your revised manuscript within **30 days Dec 23 2024 11:59PM.** If you will need more time than this to complete your revisions, please reply to this message or contact the journal office at [plospathogens@plos.org](mailto:plospathogens@plos.org). When you're ready to submit your revision, log on to <https://www.editorialmanager.com/ppathogens/> and select the **'Submissions Needing Revision**' folder to locate your manuscript file.

**Please include the following items when submitting your revised manuscript:**

* A rebuttal letter that responds to each point raised by the editor and reviewer(s). You should upload this letter as a separate file labeled 'Response to Reviewers'. This file does not need to include responses to any formatting updates and technical items listed in the 'Journal Requirements' section below.

* A marked-up copy of your manuscript that highlights changes made to the original version. You should upload this as a separate file labeled 'Revised Manuscript with Track Changes'.

* An unmarked version of your revised paper without tracked changes. You should upload this as a separate file labeled 'Manuscript'.

**Part I – Summary**

Please use this section to discuss strengths/weaknesses of study, novelty/significance, general execution and scholarship.

**Reviewer #1:** The relevant research of the manuscript findings unveils a novel, highly effective, and evolutionarily conserved, and further supplementation and modifications have been made under the suggestions and queries of the reviewers. mechanism.

**Response:** We sincerely appreciate your positive feedback on our manuscript. Thank you for your valuable insights, which have contributed to improving the quality of our work.

**Reviewer #2:** In this manuscript re-submitted to Plos Pathogens, the authors adequately addressed my criticisms by including additional experiments, yet the following minor points should be addressed prior to publication.

**Response:** Thank you for your constructive feedback. We appreciate your thorough review and your identification of minor points that still require attention. We have carefully addressed each of your concerns. We believe these adjustments have enhanced the quality of our manuscript. Thank you again for your insights, which have greatly contributed to refining our work.

**Reviewer #4:** Xue and colleagues investigated interference of IBV, a coronavirus infecting chickens, with nucleocytoplasmic trafficking of cellular transcription factors and establishment of innate antiviral responses. In brief, the authors show that IBV infection interferes with nuclear translocation of cellular transcription factors and expression of pro-inflammatory genes and genes involved in IFN responses. Furthermore, infection or directed expression of the N protein of IBV and other coronaviruses was found to alter posttranslational modification of NUP62 and other Nups and to compromise the integrity of the nuclear pore complex. In addition, the PKCα/β is shown to play a role in phosphorylation of NUP62, and RACK1 is demonstrated to be important for PKCα/β and NUP62 phosphorylation and inhibition of antiviral responses, with IBV and other coronavirus N proteins promoting p-PKCα interactions with RACK1. Finally, a nuclear export signal in IBV N protein is demonstrated to be required for suppression of innate immune responses. The results of a very comprehensive analysis are reported and the findings are of interest to the field.

**Response:** We greatly appreciate your thorough review and positive remarks regarding our manuscript.

**Part II – Major Issues: Key Experiments Required for Acceptance**

Please use this section to detail the key new experiments or modifications of existing experiments that should be absolutely required to validate study conclusions.
Generally, there should be no more than 3 such required experiments or major modifications for a "Major Revision" recommendation. If more than 3 experiments are necessary to validate the study conclusions, then you are encouraged to recommend "Reject".

**Reviewer #1:** I have no new comments for revision.

**Reviewer #2:** NONE

**Reviewer #4:** None

**Part III – Minor Issues:** Editorial and Data Presentation Modifications
Please use this section for editorial suggestions as well as relatively minor modifications of existing data that would enhance clarity.

**Reviewe****r #1:** I have no new comments for revision.

**Reviewer #2:**

1- The revised S4 Fig revealed the inhibition by other viral proteins inhibit these signaling as well as now described by the authors. Nonetheless, their results also showed that some of the viral proteins potentiate the signaling induced by PolyIC, IFN-beta TNF treatment. The authors should comment on this observation.

**Response:** Thank you for your valuable feedback on our revised S4 Fig. We appreciate your observation regarding the differential effects of viral proteins on IFN signaling pathways. We have also noted that while certain viral proteins inhibit signaling pathways, others appear to potentiate the signaling induced by Poly IC, IFNβ, and TNFα treatment. Nsp7 and nsp8 are primase enzymes that may stimulate the host's interferon response by synthesizing short RNA strands. However, the virus also encodes other proteins such as nsp15, E, M, N, 5a that reduce the host's IFN response, aiding in viral replication. The multiple regulatory strategies on host interferon response reflect the complex interplay between viral proteins and host signaling mechanisms. This regulation allows the virus to seek the best opportunities for survival within the host's immune response. We have included this discussion in the manuscript to provide clarity on this observation and its implications for our understanding of virus-host interactions. Thank you for prompting us to elaborate on this important aspect of our findings.

2- The authors should better describe how the quantification of imaging was performed relative to the nuclear staining using DAPI, i.e., how the signal intensity is calculated the in regards to the limit of the nucleus - the relative area.

**Response:** Thank you for your comment regarding the quantification of imaging. We utilized the ImageJ program (NIH, USA) in accordance with the manufacturer's instructions to quantify the intensities of the protein signals. To assess the nuclear/cytoplasmic distribution of the protein of interest, DAPI-stained nuclei were used as masks for quantifying nuclear intensity. Given that Nups and activated transcription factors are primarily localized in the nucleus, while the coronavirus N protein is found in the cytoplasm, we defined the entire cellular area based on the distribution of the N protein. This allowed us to calculate the total fluorescence intensity of the target proteins within the whole cell. We collected the total cellular fluorescence and subtracted the nuclear fluorescence of the target protein to obtain the cytoplasmic fluorescence. For each analyzed cell, the nuclear/cytoplasmic fluorescence distribution of different proteins was calculated by dividing the nuclear or cytoplasmic fluorescence by the total cell fluorescence. To ensure the reliability and accuracy of our measurements, the cells in three fields of view were analyzed for each condition. We have added this detailed description to the Materials and Methods section of the manuscript to provide clarity on our quantification approach. Thank you for highlighting this important aspect of our analysis.

**Reviewer #4:**

1- It is essential to state for all figure subpanels whether a single representative experiment or the average of several experiments is shown. If a single representative experiment is shown, please indicate how many confirmatory experiments were conducted. If the average is shown, please indicate how many experiments were averaged. Finally, please indicate whether error bars indicate SD or SEM.

**Response:** Thank you for your suggestion. For the indirect immunofluorescence analysis, we conducted three confirmatory experiments, and the representative images were shown. The quantification is based on three fields of view within a single experiment, with error bars representing standard deviation (SD) in the histogram of nuclear/cytoplasmic fluorescence distribution for the protein of interest.

For the qRT-PCR analysis, we performed three confirmatory experiments and selected one to present the results. The data are expressed as the SD of technical triplicates within one experiment. This information has been added to the Materials and Methods section.

2- The ability to interfere with Nup localization and STAT1 nuclear translocation was not only detected for the viral N protein but also for the E, M and 5a proteins. The authors might want to discuss in more detail the implications of this redundancy for IBV infection and for the specificity of the effects observed within the present study.

**Response:** Thank you for your insightful comment. We have expanded the discussion in the manuscript to highlight the implications of this redundancy for IBV infection. The specificity of the observed effects is demonstrated in the figure below, which presents three panels for proteins E, M, and 5a. Thank you for prompting us to address this important aspect of our findings.

3- Occasional style issues show be remedied: “economic animals” should read “farmed animals”. “posing a continuous threaten in poultry farms” should read “posing a continuous threat to poultry farms” “causing clinical symptoms such as vomiting and severe diarrhea, causing clinical symptoms such as vomiting and severe diarrhea” should read “causing clinical symptoms such as vomiting and severe diarrhea” “to explore the shared mechanisms utilized by pan-coronaviruses” should read “to explore the shared mechanisms utilized by diverse coronaviruses” “Initially, Vero cells, an IBV Beaudette strain adapted cell line,”. Presumably, the virus has been adapted to the cell line.

**Response:** Thank you for your feedback. We have made the necessary corrections to address the occasional style issues:

- "Economic animals" has been changed to "farmed animals."
- "Posing a continuous threaten in poultry farms" has been corrected to "posing a continuous threat to poultry farms."
- The phrase "causing clinical symptoms such as vomiting and severe diarrhea, causing clinical symptoms such as vomiting and severe diarrhea" has been streamlined to "causing clinical symptoms such as vomiting and severe diarrhea."
- All " pan-coronaviruses" have been revised to " diverse coronaviruses."
- "Initially, Vero cells, an IBV Beaudette strain adapted cell line" has been revised to “Initially, Vero cells, an adapted cell line of the IBV Beaudette strain”

All these changes have been implemented in the revised manuscript. Thank you for your suggestions.
